# Functional dynamics of dopamine synthesis during monetary reward and punishment processing

**DOI:** 10.1177/0271678X211019827

**Published:** 2021-05-30

**Authors:** Andreas Hahn, Murray B Reed, Verena Pichler, Paul Michenthaler, Lucas Rischka, Godber M Godbersen, Wolfgang Wadsak, Marcus Hacker, Rupert Lanzenberger

**Affiliations:** 1 Department of Psychiatry and Psychotherapy, Medical University of Vienna, Vienna, Austria; 2 Department of Biomedical Imaging and Image-guided Therapy, Division of Nuclear Medicine, Medical University of Vienna, Vienna, Austria; 3 Department of Pharmaceutical Sciences, Division of Pharmaceutical Chemistry, University of Vienna, Vienna, Austria; 4 Center for Biomarker Research in Medicine (CBmed), Graz, Austria

**Keywords:** Dopamine, functional PET, functional MRI, reward, sex differences

## Abstract

The assessment of dopamine release with the PET competition model is thoroughly validated but entails disadvantages for the investigation of cognitive processes. We introduce a novel approach incorporating 6-[^18^F]FDOPA uptake as index of the dynamic regulation of dopamine synthesis enzymes by neuronal firing. The feasibility of this approach is demonstrated by assessing widely described sex differences in dopamine neurotransmission. Reward processing was behaviorally investigated in 36 healthy participants, of whom 16 completed fPET and fMRI during the monetary incentive delay task. A single 50 min fPET acquisition with 6-[^18^F]FDOPA served to quantify task-specific changes in dopamine synthesis. In men monetary gain induced stronger increases in ventral striatum dopamine synthesis than loss. Interestingly, the opposite effect was discovered in women. These changes were further associated with reward (men) and punishment sensitivity (women). As expected, fMRI showed robust task-specific neuronal activation but no sex difference. Our findings provide a neurobiological basis for known behavioral sex differences in reward and punishment processing, with important implications in psychiatric disorders showing sex-specific prevalence, altered reward processing and dopamine signaling. The high temporal resolution and magnitude of task-specific changes make fPET a promising tool to investigate functional neurotransmitter dynamics during cognitive processing and in brain disorders.

## Introduction

The processing of reward and punishment represents an essential aspect of one’s mental health. This is reflected in alterations of the reward system in several psychiatric disorders such as addiction, gambling, eating disorders and depression. The prevailing approach to investigate the neural representation of behavioral effects is functional magnetic resonance imaging (fMRI) with the monetary incentive delay (MID) task being the most widely employed paradigm to study reward and punishment processing.^1^ Probing differences between monetary gain and loss consistently shows activation of the ventral striatum (VStr) including the nucleus accumbens, being a pivotal region for reward processing.^2,3^ However, blood oxygen level dependent (BOLD) fMRI is directly related to hemodynamic factors and mostly reflects post-synaptic glutamate-mediated signaling^4^ instead of mapping specific modulatory neurotransmitter action.^5^

Dopamine plays a crucial role in the processing of reward and punishment by specifically encoding these conditions. Animal research has demonstrated that the behavioral response to rewarding and aversive stimuli^6^ is mediated by different neuronal projections from the ventral tegmental area to the VStr.^7,8^ This anatomical separation also implies distinct dopamine signaling that underpin the two motivational signals. In humans endogenous dopamine release can only be assessed indirectly by specific positron emission tomography (PET) radioligands, which compete with the endogenous neurotransmitter to bind at a target receptor. Although the competition model represents a thoroughly validated approach it includes two major disadvantages when investigating human behavior. First, cognitive tasks only yield low signal changes of around 5–15% from baseline,^9^ even for a recently introduced advancement that offers high temporal resolution.^10^ Second, high specificity of observed task effects implies comparison against a control condition, but this in turn requires separate measurements. As a consequence, among those studies investigating dopamine release during monetary gain^11–14^ only one also evaluated loss, but without observing significant differences between the two conditions.^15^

An important aspect in the context of reward processing and dopamine neurotransmission is the widely described sex difference thereof. Numerous different testing schemes have shown that women are more sensitive to threats and punishment, thus aiming for risk minimization and harm avoidance. However, men tend to opt for greater rewards in terms of money, status and competitive success irrespective of the associated risks.^16,17^ Furthermore, several studies have reported general sex differences of the dopamine system, including ventral tegmental area functioning,^18^ dopamine synthesis rates at baseline^19^ and amphetamine-induced release.^20,21^ The latter has also been confirmed in rodent studies,^22,23^ but in humans this may only be present in young adults.^24^ Nevertheless, differences in reward-specific dopamine release between women and men have not yet been investigated, which is potentially attributable to the methodological difficulties mentioned above. Consequently, the neuronal underpinnings of behavioral sex differences in reward and punishment processing remain largely unknown, particularly because fMRI studies of the MID^1,25^ or other reward paradigms^26,27^ were unable to show any sex differences during reward consumption.

Therefore, the primary aim of this work was to introduce a novel approach, which enables the assessment of rapid changes in dopamine signaling during cognitive performance by extending the technique of functional PET (fPET) imaging^28,29^ to a neurotransmitter level. Here, task-induced functional dynamics of dopamine synthesis were used as an index of dopamine neurotransmission, focusing on the VStr due to its pivotal role in the processing of reward and punishment. The second aim was to demonstrate the feasibility of this technique by investigating sex differences in the processing of monetary gain and loss on a multimodal level. Thus, we combined task-induced changes in dopamine synthesis with BOLD-derived neuronal activation and modeling of behavioral data to identify the neuronal processes underlying the different behavioral sensitivity to reward and punishment in men and women.

## Theory

### Synthesis model

**Figure 1.**
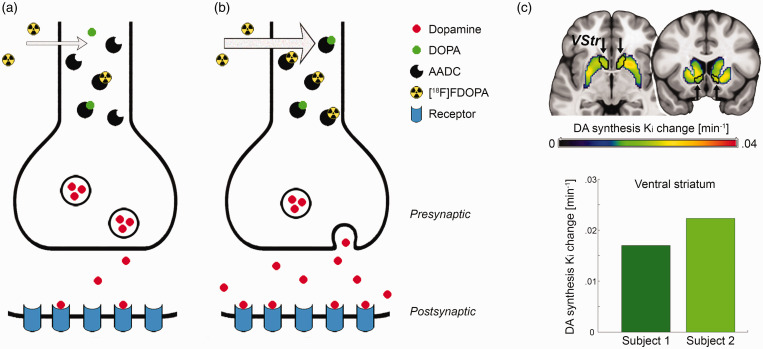
Synthesis model: (a) the neurotransmitter dopamine (DA) is synthetized from its precursor dihydroxyphenylalanine (DOPA) by the enzyme aromatic amino acid decarboxylase (AADC). Use of the radioligand 6-[^18^F]FDOPA as substrate for AADC is a well-established approach to estimate dopamine synthesis rates at baseline; (b) neuronal stimulation leads to dopamine release, but also increases AADC activity to refill synaptic vesicles with *de novo* synthetized neurotransmitter, which in turn is reflected in higher radioligand uptake as indicated by arrow thickness; (c) the proof of concept experiment showed a marked increase in striatal dopamine synthesis rates Ki during performance of the monetary incentive delay task. The ventral striatum (VStr) region of interest is outlined in black and indicated by arrows, exhibiting increases in K_i_ = 0.017 and 0.022/min from baseline for two subjects.

To assess task-relevant changes in dopamine signaling during cognitive performance we developed a novel approach, based on the dynamic regulation of neurotransmitter synthesis. As most neurotransmitters cannot pass the blood brain barrier, they are synthetized in the brain through precursor molecules. For dopamine, the main pathway is the conversion of l-tyrosine to l-3,4-dihydroxyphenylalanine (DOPA) via the enzyme tyrosine hydroxylase, and then to dopamine by aromatic amino acid decarboxylase (AADC). Importantly, these enzymes are subject to fast-acting regulatory mechanisms. Tyrosine hydroxylase and AADC activities increase with neuronal firing in order to refill the synaptic vesicles with *de novo* synthetized neurotransmitter after stimulus-induced dopamine release and are further regulated by activation or blockade of dopamine receptors.^30–34^ Moreover, the radioligand 6-[^18^F]FDOPA can be incorporated into this synthesis chain, as it is a substrate for AADC, rapidly forming 6-[^18^F]F-dopamine. The radioligand is thus specific to the dopaminergic pathway^35,36^ and represents an established approximation for dopamine synthesis rates.^34,37^ Taken together, the evidence suggests that stimulus-induced activation of dopamine synthesis is also reflected in a proportionally increased radioligand binding (Figure 1(a) and (b)).

This hypothesis can be directly tested by the application of 6-[^18^F]FDOPA within the framework of functional PET imaging.^28,38^ Similar to fMRI, fPET employs cognitive paradigms in repeated periods of task performance with an alternating control condition, thereby enabling the assessment of task-induced changes of multiple conditions within a single measurement. The radioligand 6-[^18^F]FDOPA is particularly suited for this application, as a bolus + infusion protocol^29^ further emphasizes its apparently irreversible binding characteristics^37,39^ (Suppl. Fig. S1b and e), which in turn allows to identify task-specific changes in dopamine synthesis with high temporal resolution.

## Materials and methods

### Participants

In total, 41 healthy participants were recruited for this study. Three subjects were excluded as the fPET measurement failed for technical reasons or urinary urgency. Two subjects participated in the proof of concept experiment (age 19.8 and 20.8 years, both female). The main study included 36 participants, who underwent behavioral testing with the MID task (24.5 ± 4.3 years, 18 female). Of those, 16 participants also completed fPET and fMRI examinations (24.8 ± 4.8 years, 7 female). Men and women did not differ regarding their age in the full sample (*n* = 36, p = 0.69) or the imaging subsample (*n* = 16, p = 0.51). Please see supplement for further details.

After detailed explanation of the study protocol, all participants gave written informed consent. Participants were insured and reimbursed for their participation. The study was approved by the Ethics Committee (ethics number: 2259/2017) of the Medical University of Vienna and procedures were carried out in accordance with the Declaration of Helsinki.

### Cognitive task

Reward and punishment processing was assessed using the well-established^1^ and previously employed^40^ MID task. Here, participants aim to maximize gain and avoid loss by fast reaction upon presentation of a target stimulus.

As the crucial aspect of the paradigm is the time limit of the reaction, we employed an adaptive algorithm to control the probability for gain and loss. First, the initial reaction time was individually determined directly before each testing procedure (imaging/behavior). Second, the time limit was decreased (increased) during the paradigm if the reaction was fast enough (too slow), to maintain a probability of approximately 0.5. Third, for the main study the time limit was increased (decreased) in the beginning and middle of each task block, which enabled separation of gain and loss by increasing (decreasing) the probability for each condition. The last step allowed assessment of both conditions in a single scan. Please see supplement for a detailed task description.

### Magnetic resonance imaging

MRI data was obtained on a 3T Magnetom Prisma scanner (Siemens Healthineers) using a 64 channel head coil. A structural MRI was acquired with a T1-weighted MPRAGE sequence (TE/TR = 2.29/2300 ms, voxel size = 0.94 mm isotropic, 5.3 min), which was used to exclude gross neurological abnormalities and for spatial normalization of fPET data. fMRI data was acquired using an EPI sequence (TE/TR = 30/2050 ms, voxel size = 2.1 × 2.1 × 2.8 mm + 0.7 mm slice gap).

### Positron emission tomography

The radioligand was freshly prepared every day by Iason GmbH or BSM Diagnostica GmbH. One hour before start of the fPET measurement, each participant received 150 mg carbidopa p.o. to block peripheral metabolism of the radioligand by aromatic amino acid decarboxylase.^37^ fPET imaging was carried out using an Advance PET scanner (GE Healthcare). The radioligand 6-[^18^F]FDOPA was administered in a bolus + constant infusion protocol (ratio 20:80) similar to previously described procedures^28,29^ (see supplement). During the scan the MID task was carried out at 10 (except for the PoC experiments), 20, 30 and 40 min after start of the radioligand application, each lasting for 5 min. Otherwise, a crosshair was presented and subjects were instructed to keep their eyes open and avoid focusing on anything specific (in particular not the task).

### Blood sampling

Arterial blood samples were drawn from the radial artery (see supplement). Manual samples of plasma to whole blood ratio were fitted with a linear function. Correction for radioactive metabolites was based on previous literature, assuming that the only relevant metabolite is 3-*O*-methyl-6-[^18^F]FDOPA (3-OMFD) after carbidopa pretreatment.^37,39,41^ Thus, the 3-OMFD fraction was taken from previous bolus data and modified to match our bolus + infusion protocol (see supplementary methods and Suppl. Fig. S1d). By calculation, the protocol with a bolus:infusion ratio of 20:80 indicated a 51.4% reduction of the 3-OMFD fraction (area under the curve) as compared to a pure bolus. The final arterial input function was then obtained by multiplication of the whole blood curve with the plasma to whole blood ratio and the parent fraction. The 3-OMFD input function was calculated likewise by using the 3-OMFD fraction.

### Quantification of dopamine synthesis rates

Image preprocessing was done as described previously^29^ using SPM12 and default parameters unless specified otherwise. fPET images were corrected for head motion (quality = 1, registered to mean) and the resulting mean image was coregistered to the T1-weighted structural MRI. The structural scan was spatially normalized to MNI space and the resulting transformation matrices (coregistration and normalization) were applied to the dynamic fPET data. Images were smoothed with an 8 mm Gaussian kernel, masked to include only gray matter voxels and a low-pass filter was applied with the cutoff frequency set to 2.5 min.

6-[^18^F]FDOPA time activity curves (TAC) were corrected for the 3-OMFD component using the occipital cortex as reference region. We employed a mathematical correction procedure as this avoids overcorrection compared to simple subtraction of the raw reference TAC.^42,43^ Briefly, the 6-[^18^F]FDOPA reference TAC was extracted with the Harvard-Oxford atlas and adjusted for potential task effects and movement using the same general linear model as described below. The reference TAC was fitted with a one-tissue compartment model in PMOD 3.5. The brain TAC representing the 3-OMFD component was then calculated as convolution of the 3-OMFD arterial input function with the impulse response function given by the fitted values of K_1_ and k_2_. The estimated 3-OMFD TAC was then subtracted from every brain voxel. This procedure assumes that the reference region is devoid of specific binding, that the distribution volumes of 3-OMFD and 6-[^18^F]FDOPA are equal in the reference region and that the distribution volume of 3-OMFD is uniform across the brain. A fixed whole blood component of 5% was used for all calculations.

The general linear model was used to separate task effects from baseline synthesis (Suppl. Fig. S1c). This included one regressor for each task block (except for the PoC experiments where a single task regressor was used) with a slope of 1 kBq/frame, one representing baseline dopamine synthesis and one for head motion (first principal component of the six motion regressors). As discussed in our previous work,^28^ such a task regressor assumes that task changes are constant throughout a block. The baseline was defined as average time course of all gray matter voxels, excluding those activated in the corresponding fMRI acquisition (contrast success > failure, p < 0.001 uncorrected) and those identified in a recent meta-analysis of the MID task (contrasts reward/loss anticipation and reward outcome, Suppl. Fig. S2).^1^ The Gjedde–Patlak plot was then applied to compute the net influx constant K_i_ as index of dopamine synthesis for baseline and task effects separately (Suppl. Fig. S1e). The slope was fitted from t* = 25 min, which is half of the scan time. The four task blocks were finally weighted according to task performance (actual gain/possible gain, similar for loss) and averaged to obtain task specific K_i_ for gain and loss. To assess the specificity of the findings, task-specific changes in dopamine synthesis rates were also calculated as percent signal change from baseline with

(1) $${\text{PSC}}_{\text{Ki}}={\text{Ki}}_{\text{task}}/{\text{Ki}}_{\text{baseline}}*\text{1}00$$

and without weighting by task performance.

### Neuronal activation

Task-induced neuronal activation was computed as described previously using SPM12.^40^ fMRI BOLD images were corrected for slice timing differences (reference: middle slice) and head motion (quality = 1, registered to mean), spatially normalized to MNI space and smoothed with an 8 mm Gaussian kernel. Neuronal activation was estimated across the two runs with the general linear model including one regressor for each cue (gain, loss, neutral), one for the target stimulus and one for each of the potential outcomes (gain, omitted gain, loss, avoided loss, neutral) as well as several nuisance regressors (motion, white matter, cerebrospinal fluid). To obtain an index of reward outcome^1^ which is as similar to fPET as possible, parameter estimates were combined as (gain + avoided loss) – (omitted gain + loss). Percent signal changes were computed as

(2) $${\text{PSC}}_{\text{fMRI}}=\beta_{\text{task}}/\beta_{\text{baseline}}*\text{1}00*\text{peak}$$

with *β*_baseline_ and peak representing the constant and the peak value of the fMRI design matrix, respectively.^44^

### Statistical analysis

All statistical tests were two-sided and corrected for multiple comparisons with the Bonferroni-Holm procedure (e.g., when testing multiple conditions and/or groups) and the reported p-values have been adjusted accordingly.

For behavioral data, the accumulated amount of money that was gained and lost during the corresponding task blocks of the MID were assessed with one sample t-tests against zero, whereas sex differences were computed by independent samples t-tests. Due to the adaptive nature of the MID task the reaction times were normalized to the mean by subtracting the average reaction time within each block. Differences in reaction times were evaluated by repeated measures ANOVA with the factors sex and amount. Post-hoc t-tests were used to assess sex differences for each amount of money. Furthermore, we modeled the relationship between reaction time and amount with a stepwise linear regression up to second-order polynomial functions. Stepwise regression choses the model that best explains the data based on statistical significance. This was done across the entire group (*n* = 36) to test for a general relationship. Subsequently, parameters of the resulting models were also estimated individually for the fPET subjects (*n* = 16) to assess the correlation with task-specific changes in dopamine synthesis using Spearman’s correlation (since *n* < 10 in each group for fPET).

For imaging parameters, the primary region of interest was the VStr due to its pivotal importance in reward processing.^2,3^ Therefore, values of K_i_ and PSC_fMRI_ were extracted for this region using the Harvard Oxford atlas as provided in FSL (termed “nucleus accumbens” in the atlas). For comparison, a functional definition of the VStr was also employed (neuronal activation of reward outcome^1^ within the striatum), which comprised 2.55 cm^3^ (in contrast to the nucleus accumbens of the Harvard-Oxford atlas with only 1.38 cm^3^). Task-specific changes in dopamine synthesis rates were evaluated by one sample t-tests against zero for gain and loss separately. Similarly, for K_i_ and PSC_fMRI_ the difference of gain vs. loss was calculated and assessed by one sample t-tests against zero (i.e., being identical to a paired samples t-tests). Finally, sex differences in K_i_ and PSC_fMRI_ were addressed using an independent samples t-test.

In an exploratory analysis K_i_ values were extracted from the caudate and putamen as defined by the Harvard-Oxford atlas and investigated in the same manner as the VStr.

## Results

### Proof of concept

To assess the feasibility of the proposed synthesis model an initial PoC experiment was conducted. Two subjects underwent fPET imaging with the radioligand 6-[^18^F]FDOPA while performing the MID task. In both subjects the task induced substantial increases in VStr dopamine synthesis of K_i_ = 0.017 and 0.022/min from baseline (Figure 1(c)), supporting the feasibility of the approach to assess task-specific changes in dopamine neurotransmission.

### Behavioral data

Since the PoC experiment combined monetary gain and loss within a task block, the main study specifically aimed to disentangle these two effects on a behavioral (*n* = 36) and neurobiological level (*n* = 16, see below). The task was extended to four blocks and each of them manipulated to enable the separate assessment of monetary gain and loss.

**Figure 2.**
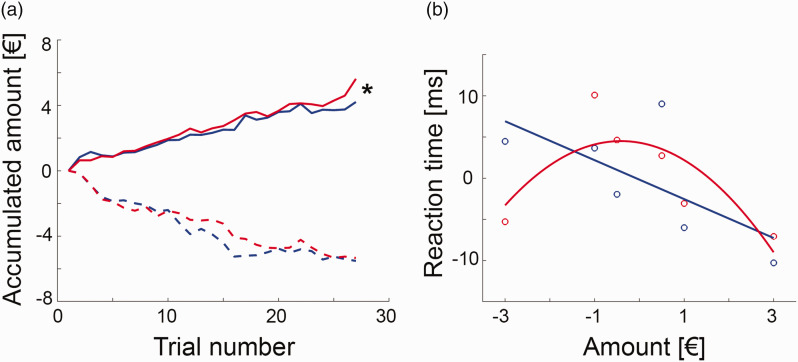
Behavioral data (blue = men, red = women): (a) the monetary incentive delay task was manipulated by modifying the reaction time limit for a successful trial completion, which enabled separate assessment of monetary gain and loss. During the gain task block women earned significantly more money than men (5.6 ± 2.4 € vs. 4.2 ± 1.7 €, t = 2.1, *p = 0.047), but both groups showed similar loss (–5.3 ± 1.9 € vs. –5.5 ± 1.9 €, p = 0.8). Lines represent average accumulated monetary amount at each trial; (b) the association between the individually normalized reaction times and the gained/lost amount of money was modelled by a linear relationship in men (reaction time = –0.18 – 2.36 * amount, p_linear_ = 0.0012, p_quadratic_ = 0.11), where a steeper negative slope indicated faster reaction time and thus higher sensitivity for reward. In contrast, the association was characterized by an inverted U-shaped function in women (reaction time = 4.31 – 0.95 * amount – 1.16 * amount^2^, p_linear_ = 0.2, p_quadratic_ = 0.001). Since these two functions exhibit the most pronounced difference for high amounts of loss, a strong negative quadratic term was interpreted as high sensitivity for punishment. Circles denote average values for each amount and lines are model fits across the entire data set (*n* = 18 women and 18 men, see Suppl. Fig. S3 for details), reaction times are mean centered due to the adaptive nature of the MID task.

Behavioral data showed that average monetary gain and loss were significantly different from zero (all t = 10.0 to 12.6, p = 1.7 × 10^−8^ to 2 × 10^−9^, Figure 2(a)), indicating successful task manipulation. Women gained significantly more than men (5.6 ± 2.4 € vs. 4.2 ± 1.7 €, t = 2.1, p = 0.047), but both groups showed similar loss (–5.3 ± 1.9 € vs. –5.5 ± 1.9 €, p = 0.8).

The difference in monetary gain was also reflected in the normalized reaction times, with a main effect of sex (F_(1,34)_ = 6.9, p = 0.013) and amount (F_(5,170)_ = 4.4, p < 0.001) as well as a trend for an interaction effect sex * amount (F_(5,170)_ = 2.0, p = 0.08). Post-hoc t-test indicated that this seemed to be driven by the –3 € condition with women showing a faster reaction than men (t = 2.0, p = 0.049).

We further aimed to model the behavioral response in more detail, as the relationship between reaction time and amount for each group. In men, this was best described by a negative linear function (reaction time = –0.18 – 2.36 * amount, p_linear_ = 0.0012, p_quadratic_ = 0.11), with a faster reaction for higher monetary gains (Figure 2(b), Suppl. Fig S3). In contrast, the association in women was characterized by an inverted u-shaped function (reaction time = 4.31 – 0.95 * amount – 1.16 * amount^2^, p_linear_ = 0.2, p_quadratic_ < 0.001), with faster reaction times for high amounts of loss as compared to men. These distinct relationships for men and women were also obtained for the imaging subsample (men p_linear_ = 0.009, p_quadratic_ = 0.08; women: p_linear_ = 0.7, p_quadratic_ = 0.03). Thus, we interpreted the linear (quadratic) term for men (women) as index for reward (punishment) sensitivity, that is, the more negative the parameter, the faster the reaction time for high gain (loss).

### Functional dynamics in dopamine synthesis

**Figure 3.**
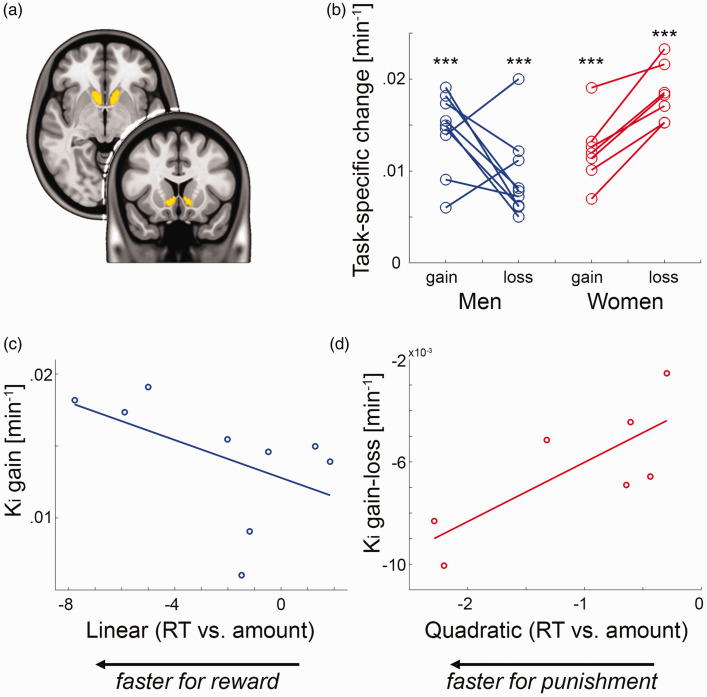
Functional PET imaging of task-specific dopamine synthesis: (a) region of interest of the ventral striatum (VStr) from the Harvard-Oxford atlas; (b) processing of monetary gain and loss resulted in pronounced increases in VStr dopamine synthesis K_i_ (***all p < 0.001). While men showed higher dopamine synthesis changes for gain vs. loss (*n* = 9), women exhibited the opposite pattern (*n* = 7, see also Figure 4(a)); (c,d) the individually modelled associations between reaction time (RT) and amount were used as indices for reward and punishment sensitivity in men (linear term) and women (quadratic term), respectively (see Figure 2(b)). These behavioral indices showed an association with task-specific changes in VStr dopamine synthesis during monetary gain in men (c) *ρ* = –0.67, p = 0.059) and the difference between gain and loss in women (d) *ρ* = 0.79, p = 0.048).

**Figure 4.**
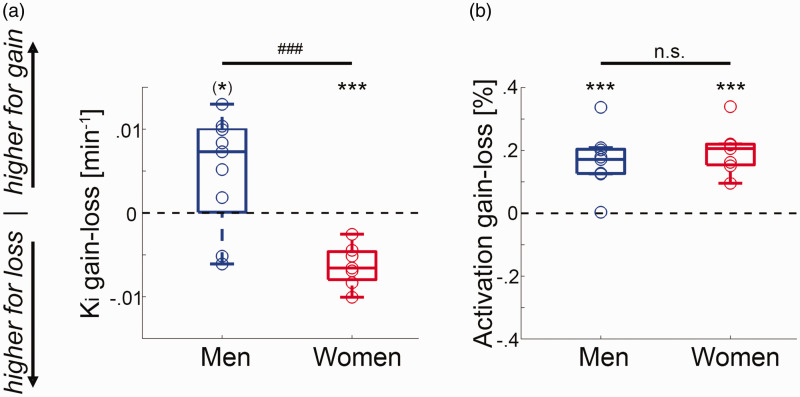
Comparison between fPET and fMRI: (a) in men the task-specific changes in VStr dopamine synthesis K_i_ were higher for gain than for loss (^(^*^)^p = 0.06). In contrast, women showed the opposite pattern with higher changes in dopamine synthesis during loss vs. gain (***p < 0.001), leading to a significant difference between the two groups (t = 4.1, ^###^p < 0.001); (b) although neuronal activation obtained with BOLD fMRI indeed showed robust VStr signal changes for the contrast gain versus loss for men and women (t = 5.5 to 6.9, ***p = 0.0005 to 0.0006), there was no significant difference between the two groups (p = 0.4). Boxplots indicate median values (center line), upper and lower quartiles (box limits) and 1.5 * interquartile range (whiskers).

To assess reward-specific changes in dopamine synthesis, 16 of the above subjects also underwent fPET with the radioligand 6-[^18^F]FDOPA (seven female). The MID task yielded increased dopamine synthesis rates in the VStr during gain (men: K_i_ = 0.014 ± 0.004/min, women: K_i_ = 0.012 ± 0.004/min) and loss (men: K_i_ = 0.009 ± 0.005/min, women: K_i_ = 0.019 ± 0.003/min, all t = 6.0 to 16.1, all p < 0.001, Figure 3(b)

). This corresponds to changes from baseline K_i_ in the range of 105 ± 31% to 165 ± 64%. As a result, the direct comparison between the two conditions showed higher dopamine synthesis rates in men for gain vs. loss (K_i_ = 0.005 ± 0.007/min = 59 ± 77% from baseline, t = 2.2, p = 0.06). Interestingly, the direction of this difference was reversed in women with higher dopamine synthesis during loss vs. gain (K_i_ = –0.006 ± 0.003/min = –55 ± 25% compared to baseline, t = –6.6, p < 0.001, Figure 4(a)).

Exploratory assessment of differences between the first and second task blocks, showed a trendwise increase in dopamine synthesis for gain in men (p = 0.05). However, no such difference was observed for women or the loss condition for both sexes (all p > 0.25), indicating no systematic influence of the task timing on dopamine synthesis.

Proceeding from the distinct models to characterize the behavioral response of monetary gain and loss (Figure 2(b)), we assessed the relationship between individual model parameters (linear and quadratic terms) and task-specific dopamine synthesis rates. This resulted in an association in men between the linear term and VStr dopamine synthesis during gain (*n* = 9, *ρ* = –0.67, p = 0.059, Figure 3(c)). On the other hand, the quadratic term in women was positively associated with VStr dopamine synthesis of gain vs. loss (*n* = 7, *ρ* = 0.79, p = 0.048, Figure 3(d)).

### Sex differences

Finally, VStr dopamine synthesis rates between gain vs. loss were significantly higher for men than women (t = 4.1, p < 0.001, Figure 4(a)). This sex difference was similarly present when using a functional delineation of the VStr ^1^ (t = 3.9, p = 0.002), for percent signal change from baseline (t = 3.8, p = 0.002) and without weighting by task performance (t = 3.1, p = 0.009).

Exploratory analysis showed a sex difference for the putamen (t = 3.3, p = 0.005). This was driven by higher dopamine synthesis for loss than gain in women (t = –3.3, p = 0.03), as men did not show any difference between the two conditions (p = 0.2, Suppl. Fig. S4). The caudate indicated no sex difference and also no difference between gain and loss (all p > 0.1). Furthermore, no significant associations between synthesis rates and behavioral values for putamen and caudate were found (all p > 0.2).

For direct comparison we also assessed neuronal activation, where the same subjects as in the fPET experiment also underwent fMRI. In line with previous reports^1^ we observed robust neuronal activation in the VStr for gain vs. loss in men and women (t = 5.5 to 6.9, all p < 0.001, Figure 4(b)). As expected, there was however no significant sex difference in activation for the atlas-based (p = 0.4) or the functional delineation of the VStr (p = 0.3).

Similar to another study,^19^ our method was able to replicate previously observed sex differences in *baseline* VStr dopamine synthesis (men: K_i_ = 0.009 ± 0.001/min, women: K_i_ = 0.012 ± 0.002/min, t = 3.0, p = 0.009). It is however unlikely that these baseline differences affect the task-specific estimates (see limitations).

## Discussion

In this work we introduce a novel framework for the assessment of task-specific changes in dopamine neurotransmission, which is based on the dynamic regulation of neurotransmitter synthesis quantified by functional PET imaging. Processing of monetary gain and loss induced robust changes in dopamine signaling in the living human brain even for the direct comparison of these two conditions, demonstrating the high sensitivity and specificity of the approach. Crucially, task-induced changes in dopamine synthesis showed sex-specific differences in the opposite direction with higher synthesis rates in men for gain vs. loss but vice versa in women, directly reflecting behavioral sex differences in reward and punishment sensitivity. Since this sex difference was not present in common BOLD-derived assessment of neuronal activation, our findings have important implications for the interpretation of numerous fMRI studies on reward processing. This is also essential in various clinical populations, where the sex-specific influence on the link between altered reward processing and dopamine signaling is not yet fully understood.^45^

The current work provides a biological basis for the well-known behavioral differences in reward and punishment sensitivity between men and women.^16,17^ We hereby extend general sex differences of the dopamine system^19–21^ specifically to the processing of gain and loss and directly link changes in dopamine neurotransmission with the corresponding behavioral response. This is also supported by pharmacological effects observed in animals and humans. For instance, male rats aim for large rewards independent of the risk, whereas females decrease such choices in order to avoid punishment. This sex difference was even more pronounced by the dopamine releasing agent amphetamine, where females abolished the choice for risky rewards to a much larger extent than males.^46^ On the other hand, studies in humans have shown that men often opt for selfish rewards, but women take more prosocial choices. However, pharmacological blockade of dopamine D2/D3 receptors shifts these preferences and thereby eliminate the sex difference in prosocial choices, i.e., men and women showed similar preference for selfish rewards.^47^ Taken together, these findings suggest that sex differences in reward behavior are substantially driven by dopamine neurotransmission. It is worth to note that pharmacological challenges may represent an unspecific assessment of neurotransmitter action. The systemic manipulation affects the entire brain, possibly eliciting complex downstream effects, and the use of potent challenge agents may overshadow subtle physiological and behavioral differences. Therefore, our results provide novel evidence in this context through the direct and spatially targeted assessment of reward-specific dopamine signaling itself, without manipulation of the neurotransmitter system. This enabled us to disentangle the dopaminergic involvement in monetary gain and loss, which revealed opposing changes in synthesis rates between men and women.

In contrast, such an evaluation was not accessible by previous approaches (see introduction for PET findings on the competition model), including reward-specific neuronal activation obtained with fMRI. Again, it needs to be emphasized that neither this nor other fMRI studies^1,25,26^ revealed any (and particularly not opposing) sex differences in VStr activation between gain and loss. fMRI based on the BOLD signal is dependent on the link between neuronal activation and changes in hemodynamic factors such as blood flow, volume and oxygenation.^5,48^ Blood flow is locally controlled by the major neurotransmitter glutamate, and thus it is widely accepted that the BOLD signal mostly reflects postsynaptic glutamate-mediated signaling.^4,49^ Although monoamine neurotransmitters such as dopamine may also modulate blood flow,^50^ this does not seem to translate into corresponding fMRI signal changes, at least for the processing of monetary gain and loss using the widely employed MID task. We acknowledge that previous work has indicated a relationship between dopamine release and fMRI,^51,52^ but these were again based on potent pharmacological manipulations, which may not be directly comparable to more subtle cognitive effects (see above). Instead, it appears that during cognitive task performance the limited contribution of dopamine to the BOLD signal gets lost in major downstream effects of glutamate action^4^ that regulate blood flow. We speculate that the latter two are not sufficiently specific^5^ to identify sex differences in neuronal activation during reward processing. This may have substantial implications for the investigation of several brain disorders with dopamine dysfunction such as addiction, schizophrenia or depression, where fMRI represents one of the most widely used methods. Our results suggest that BOLD signal alterations may not primarily reflect the underlying dopaminergic changes, especially when investigating the reward system in men and women. Further work is required to elucidate the exact difference that cognitive and pharmacological stimulation exert on the relationship between BOLD imaging and dopamine signaling and if this extends beyond sex differences of reward processing.

It also needs to be highlighted that the reward circuit goes beyond the VStr and includes numerous other brain regions such as the frontal cortex and midbrain areas of the substantia nigra, ventral tegmental area and raphe nuclei. In particular, the VStr receives inputs from medial prefrontal, orbitofrontal and dorsal anterior cingulate cortices as well as the amygdala, which mediate reward behavior.^3^ The involvement of the raphe nuclei also implies a substantial contribution of the serotonin system in the processing of aversive and rewarding stimuli.^53^ This is further supported by changes in reward behavior and neuronal activation after antidepressant treatment that target the serotonin system.^54^ Therefore, future work may aim to elucidate the interaction of VStr dopamine signaling with other brain regions and neurotransmitters during reward processing.

On the other hand, we also observed task-specific dopamine synthesis in the putamen and caudate. This is in line with previous work on motor tasks and cognitive processes,^9,55^ considering that the MID paradigm also requires a fast motor response. However, the sex difference between gain and loss in the putamen was driven by women, which was less pronounced than in the VStr and no associations with the behavioral response were found.

Although not directly assessed, there are two essential lines of evidence which strongly support the concept that task-specific changes in the 6-[^18^F]FDOPA signal are related to dopamine release. As mentioned, dopamine synthesis is subject to fast-acting regulatory mechanisms, which is activated by neuronal firing to refill the synaptic vesicles.^30–32^ Moreover, dopamine synthesis is also increased by the dopamine releasing agent amphetamine as demonstrated in rats^56^ and monkeys using PET.^57^ In a similar manner decreasing dopamine synthesis also decreases amphetamine-induced dopamine release.^58,59^ Notably, a previous study reported no relationship between dopamine synthesis and release,^60^ but it is important to mention that synthesis was only investigated at baseline (i.e., without any task- or drug-induced stimulation). In contrast, we specifically assessed changes in dopamine synthesis during task performance and thus the previous finding is not in contrast to the synthesis model. Hence, the herein proposed approach offers an alternative to the competition model as the crucial factor to identify task-specific changes is the incorporation of radioligands into the dynamic regulation of enzymes responsible for neurotransmitter synthesis (instead of direct competition between radioligand and endogenous neurotransmitter).

The different neurobiological basis of these two approaches (i.e., competition vs. synthesis model) seems to explain the marked signal changes observed during the reward task for the comparison against baseline and for gain vs. loss. This underlines the high sensitivity of the technique but also the high specificity, with the ability to separate subtle effects of behaviorally similar conditions. Furthermore, fPET allows to assess task-specific changes of multiple conditions in a single within-scan design, thereby eliminating intrasubject variability related to differences in habituation, motivation or performance of repeated measurements. These advantages seem to translate into robust effects even with a low sample size, thereby mitigating the limitation of the current study that imaging was only performed in a subset of the cohort.

Of note, task-specific changes in K_i_ appear rather high, with a 100–165% increase from baseline indicating an estimated 150–285% increase in k_3_ (presumably reflecting AADC, see supplement, quantification of dopamine synthesis). Although simulations suggest that dopamine synthesis can increase up to five-fold,^61^ changes in AADC activity will not equally translate into storage or release of dopamine. It has been shown that 75–90% of DOPA is available for dopamine synthesis in rats, however this estimate was only 50% for humans.^62,63^ Furthermore, from this fraction another 25% of dopamine is metabolized and thus not stored in vesicles.^61^ Together, this suggests approximately 56–107% of additionally synthesized dopamine by task performance. This is well within the physiological range of dopamine release in rats during reward and punishment.^64^ Nevertheless, further work is required to determine the exact relationship between changes in 6-[^18^F]FDOPA signal as index of dopamine synthesis and its release, as these processes are tightly coupled.^56–59^

Another limitation is the use of a literature-based correction for radioactive metabolites instead of an individual one. Although this may indeed change the absolute values of dopamine synthesis to a certain extent, it does not influence the reward-specific effects. Again, in a within-scan design any “global” parameter will affect baseline and task-specific synthesis rates in an equal manner and will thus cancel out when calculating percent signal change or differences between gain and loss. This applies for instance to radioactive metabolites as well as sex differences in dopamine synthesis at baseline.^19^ Interestingly, recent work indicated generally lower dopamine uptake in women than men in the putamen.^65^ However, for the specific age range of subjects included in the current study (third decade) this effect was actually reversed, which concurs with our findings. Irrespective of the direction of this effect, baseline differences (if at all) would most likely cause general differences in task-specific dopamine synthesis across all task conditions. However, the observed task-specific changes were higher in men than women for gain, but vice versa for loss, which argues against a dependency of task estimates on baseline synthesis.

Finally, further work is required to confirm the linear and quadratic relationships of reward-related reaction times in men and women, respectively, and the associations with dopamine synthesis.

To summarize, the current work provides a strong motivation for further investigations of functional neurotransmitter dynamics during cognitive processing. The framework of fPET imaging offers important advantages of high temporal resolution, robust effect size of task-induced changes and the possibility to assess multiple task conditions in a single measurement. Future studies should aim for an in-depth evaluation of stimulus-dependent activation of dopamine synthesis, proceeding from previous findings which link neurotransmitter synthesis and release.^56,57^ Moreover, our results suggest that reward-specific neuronal activation should not unequivocally be interpreted as corresponding changes in dopamine signaling and that the investigation of sex differences in this context requires further attention. This may be of pivotal relevance for the assessment of numerous psychiatric and neurological patient populations. These include for instance addictive, gambling and eating disorders or depression as well as autism spectrum disorder and Parkinson’s disease, given the different prevalence rates in men and women as well as alterations in reward processing and dopamine signaling.^66–71^ The introduced approach enables to address important future questions of human cognition and to investigate whether the observed reward- and sex-specific differences in dopamine synthesis will translate to clinically relevant characteristics for patient diagnosis or treatment.
