# Supplementary material for: Functional dynamics of dopamine synthesis during monetary reward and punishment processing
Source: J Cereb Blood Flow Metab. 2021 May 30;41(11):2973–85. doi: 10.1177/0271678X211019827 (PMC8543667; doi:10.1177/0271678X211019827)
Supplement: Supplementary file 1 [file sj-pdf-1-jcb-10.1177_0271678X211019827.pdf]

SUPPLEMENTARY MATERIAL FOR

Functional dynamics of dopamine synthesis during monetary reward and punishment processing

Andreas Hahn[1*], Murray B. Reed[1], Verena Pichler[2], Paul Michenthaler[1], Lucas Rischka[1], Godber M. Godbersen[1], Wolfgang Wadsak[2,3], Marcus Hacker[2], Rupert Lanzenberger[1*]

[1] *Department of Psychiatry and Psychotherapy, Medical University of Vienna, Austria*
[2] *Department of Biomedical Imaging and Image-guided Therapy, Division of Nuclear Medicine, Medical University of Vienna, Austria*
[3] *Center for Biomarker Research in Medicine (CBmed), Graz, Austria*

[*] **Correspondence to:**

Andreas Hahn, Prof. PD PhD MSc
Email: andreas.hahn@meduniwien.ac.at
ORCID: 0000-0001-9727-7580
or
Rupert Lanzenberger, Prof. PD MD
Email: rupert.lanzenberger@meduniwien.ac.at
ORCID: 0000-0003-4641-9539

Department of Psychiatry and Psychotherapy
Medical University of Vienna, Austria
Waehringer Guertel 18-20, 1090 Vienna, Austria

MATERIALS and METHODS

Participants

General health of all participants was ensured at the screening visit by an experienced psychiatrist as based on the subjects' medical history. Exclusion criteria were current and previous (12 months) somatic, neurological or psychiatric disorders, current and previous substance abuse or psychotropic medication. For participants who underwent fPET and fMRI a more extensive medical examination was carried out, including blood tests, electrocardiography, neurological testing and the Structural Clinical Interview for DSM-IV. Additional exclusion criteria were contraindications for MRI scanning, previous study-related radiation exposure (10 years), pregnancy or breast feeding. Thus, female participants underwent a urine pregnancy test at the screening visit and before the fPET and MRI scans.

Cognitive task

The task was designed in an event-related manner. Each trial started with the presentation of the potential gain or loss (e.g., +3 €, -1 €) for an unknown variable duration of 3 - 5 s (0.5 s steps, uniformly distributed). After that the target stimulus (!) was shown and subjects were required to press a button as fast as possible. If the reaction was within a given time limit the amount was gained or loss was avoided. Otherwise, the amount was not gained or lost. Each button press was followed by immediate feedback (2 s), showing the amount gained or lost, the outcome of the reaction (in green for success, red for failure) and the current account sum. Trials were separated by a crosshair with a variable duration of 3 - 7 s (1 s steps, uniformly distributed). To maintain a high level of attention and to enable modeling of the behavioral response, 6 different amounts of money were used for the task (0.5, 1 and 3 € each for gain and loss, initial amount was 10 €). Furthermore, motivation of the participants was kept high by the instruction that the final amount was paid out in addition to a fixed reimbursement.

The crucial aspect of this paradigm is the time limit of the reaction for the button press, where we employed an adaptive algorithm. For fPET and behavioral testing the task was carried out

in several blocks, thus, the initial time limit of each block was set to the median of the reaction times of the previous block. The time limit for the first block (and for fMRI) was determined as median of 8 trials taken right before the start of the experiment in the scanner. Second, the time limit for each of the different amounts was adaptively decreased (increased) within a task block by 50 ms if the reaction was fast enough (too slow). These settings maintain an average probability of approximately 0.5 to gain or lose a certain amount.

For the proof of concept (PoC) experiments, 3 task blocks of 5 min were carried out each with 27 trials (equal distribution of amounts presented in random order) without further manipulation of the reaction time limits (i.e., probability of 0.5 for gain and loss, Suppl. Fig. S1a).
For the main study, 4 task blocks of 5 min were carried out in a similar manner, but the probability for monetary gain and loss was manipulated within each block by changing the reaction time limit (Suppl. Fig. S1a). For each of the 2 blocks of monetary gain (loss), the initial time limits of all amounts were increased (decreased) by 50 ms. Furthermore, in the middle of each block the time limits were reset to the median of the preceding reaction times and 25 ms were again added (subtracted). This enabled the separate assessment of gain and loss even within the lower temporal resolution of fPET. The two conditions were presented in alternating order with randomization of the starting condition. The order of the task blocks was counterbalanced between men and women (n=4 with order gain-loss-gain-loss in each group). Of note, monetary "gain" in this setting combines actual gain with avoided loss, whereas "loss" represents actual loss and omitted gain. The requirement for such a design is however well supported by previous studies indicating that avoided loss represents a relative reward [1]. For the behavioral testing, participants completed the same task version as for fPET but outside the scanner. For the fMRI measurements the MID task was carried out in 2 runs of 8.2 min each. The manipulation of the time limits (i.e., probability for gain and loss) was similar to that of fPET, but the trial order was randomized to avoid blocks of continuous gain or loss since fMRI has a higher temporal resolution and allows to model each trial separately. Furthermore, a neutral condition was included where no money was at stake (0 €). For all task versions,

participants were blind to the adaption and manipulation of the time limits. This was also ensured by the design, where the probability for gain and loss is individually controlled via the time limit, since overruling the participant's actual reaction time would be easily recognized. The task was implemented in Psychtoolbox v3.0.12.

Positron emission tomography (PET)

To minimize head movement each participant's head was placed in a cushioned polyurethane bowl with straps around the forehead. The paradigm was visualized on a common LCD screen and presented to the subjects by a mirror, which was placed in front of the participant's eyes using a custom-made wooden construction. Attenuation correction was performed for 5 min with retractable ^{68}Ge rod sources, which also included the mirror construction. Dynamic fPET acquisition in 3D-mode started with the intravenous bolus + infusion of the radioligand 6-[^{18}F]FDOPA. The injected dose was 5.5 MBq/kg body weight and the measurement time was 50 min. To increase the signal to noise ratio, 20% of the dose was given as bolus for 1 min [2] and the remainder as constant infusion for the rest of the scan (89 kBq/kg/min) using a perfusion pump (Syramed µSP6000, Arcomed, Regensdorf, Switzerland). fPET images were then reconstructed to frames of 43 s, yielding 70 frames in total and 7 frames for each task block.

Blood sampling

Automatic sampling was carried out for the first 5 min (4 ml/min, Allogg, Mariefred, Sweden). Manual samples were taken at 3, 4, 5, 16, 26, 36 and 46 min, i.e., at periods of task pauses. For one subject only manual samples were available. For the manually obtained samples, activity in whole blood and plasma (after centrifugation) were measured in a gamma counter (Wizward^2, 3", Perkin Elmer). Automatic and manual samples were then combined, were the first manual samples served for a measurement-specific calibration between the automated sampling system and the gamma counter.

The 3-OMFD fraction was not measured in this study but extracted from previous work [3, 4] and fitted with a single exponential function (Suppl. Fig. S1d). Since this represents the metabolite fraction after a bolus application, we adapted the function to the herein employed radioligand application protocol as the sum of an initial 20% bolus and proportionally lower boli administered every further minute (Suppl. Fig. S1d). We are aware that a literature-based metabolite correction may affect the individual estimates of dopamine synthesis rates. However, it equally affects task-specific changes thereof as they are acquired within the same measurement. Thus, the individual variation in metabolism will cancel out when calculating percent signal change from baseline and differences between task conditions (see also discussion). Moreover, the bolus + infusion protocol resulted in a reduction of the 3-OMFD fraction by 51.4% (area under the curve), further minimizing the influence of radioactive metabolites.

Quantification of dopamine synthesis rates

Based on available literature values we aimed to put our results in relation to changes in AADC activity and dopamine release. The Patlak plot yields the net influx constant $K_i = K_1 * k_3/(k_2+k_3)$ as an index of dopamine synthesis, whereas the rate constant k_3 presumably represents AADC activity. Assuming that task-specific changes in K_i are attributable to variation in k_3, the change in AADC activity during reward processing can be estimated. We therefore extracted baseline TACs for the VStr after removal of task effects with the GLM. The TACs were then modelled using a two-tissue compartment model in PMOD with $k_4 = 0$. This resulted in average values across all subjects of $K_1 = 0.042$ ml*cm^{-3}*min^{-1}, $k_2 = 0.064$ min^{-1} and $k_3 = 0.012$ min^{-1}, yielding $K_i = 0.0066$ min^{-1}. We then entered these values in the equation above, constrained K_1 and k_2 to remain unchanged and varied k_3 to obtain changes in K_i as observed in the current study. Thus, the increase in task K_i of 100-165% from baseline suggests an additional 150-285% (i.e., 2.5 to 3.85-fold increase) in k_3.

Statistical analysis

To rule out a potential bias in the definition of the baseline term in the 6-[^{18}F]FDOPA GLM analysis, the voxels included in this regressor were compared between women and men (Suppl. Fig. S2). For each group a separate mask was created, where each voxel indicates the number of subjects that use that particular voxel for the baseline definition. These masks were binarized at 50% of subjects per group and compared between men and women using the Dice coefficient.

SUPPLEMENTARY FIGURES

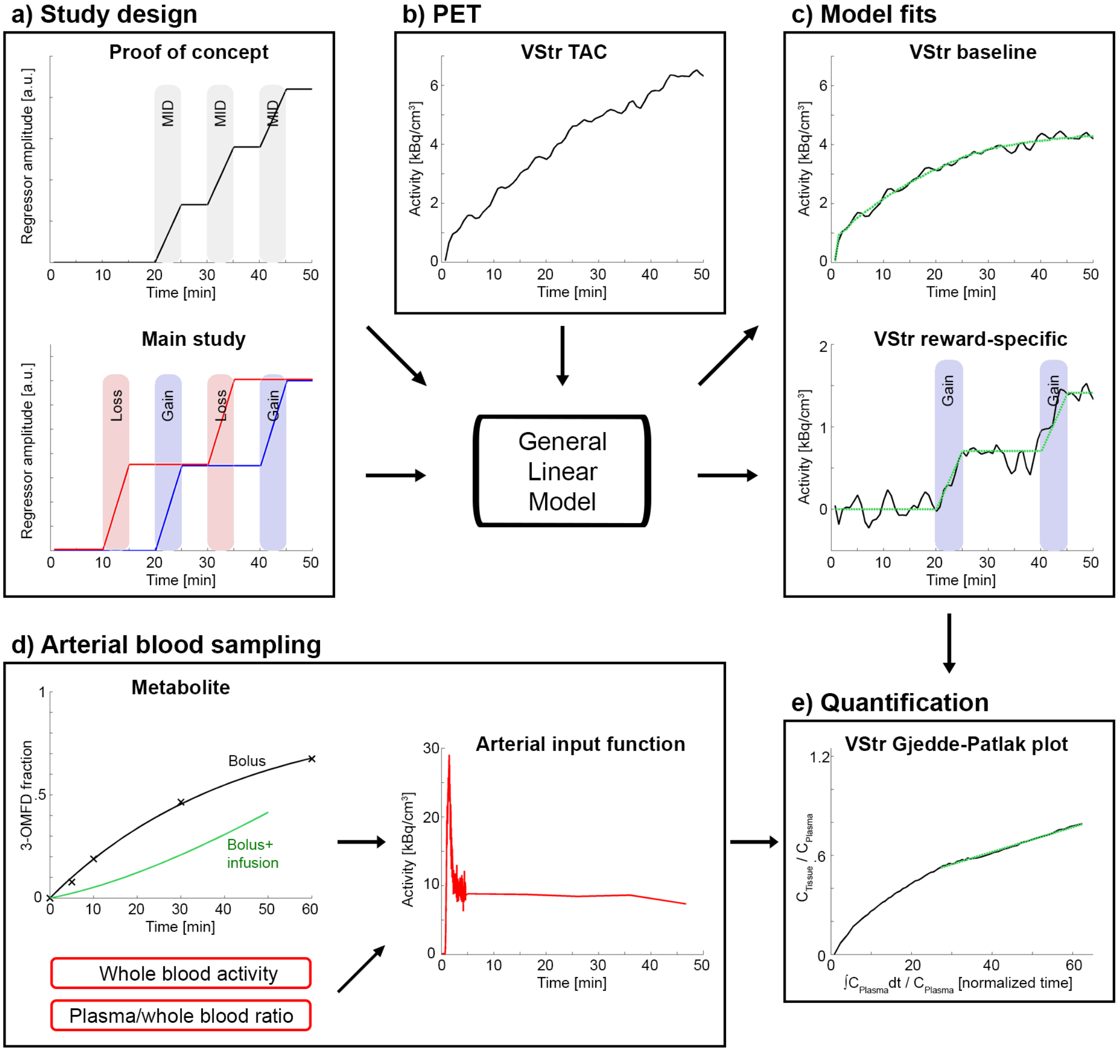

Supplementary figure S1: *fPET analysis workflow*. **a)** During the PET scan the monetary incentive delay (MID) task was carried out in blocks of 5 min. In the proof of concept experiment 3 blocks were employed without a separation of gain and loss conditions and an average probability for success of 0.5. For the main study 4 blocks were completed and the MID task was manipulated to disentangle gain and loss by increasing (decreasing) the probability for success (failure) within the corresponding blocks. The order of gain and loss blocks was alternating for both groups and counterbalanced between men and women. **b)** PET measurements were carried out using the radioligand 6-[^{18}F]FDOPA. Time activity

curves (TAC) were corrected for the radioactive metabolite 3-OMFD with a mathematical correction method [5, 6]. The bolus + infusion protocol emphasized the irreversibly uptake of the radioligand. **c)** TACs were then modeled according to the study design to separate baseline and task-specific effects. Model fits (green dotted line) indicate a robust increase in radioligand uptake during the task condition in the ventral striatum (VStr). **d)** The metabolite fraction (3-OMFD) was extracted from previous studies [3, 4] (black crosses), fitted with a single exponential function (black line) and adapted to match the bolus + infusion protocol (green line). This was combined with the individually measured whole blood activity and plasma to whole blood ratio to obtain the arterial input function, which reaches steady state approximately after 5 min. **e)** Quantification was carried out with the Gjedde-Patlak plot from t* = 25 min onwards, yielding dopamine synthesis rates at baseline and for each task condition. All data for the TAC (b), model fits (c), arterial input function (d) and quantification (e) were extracted from a representative subject. Metabolite data for a bolus application (d, black) as extracted from the literature [3, 4] were 7.8, 19.0, 46.5 and 67.5% at 5, 10, 30 and 60 min. The metabolite data for the bolus + infusion protocol (d, green) as used in this study were 2.3, 5.2, 21.0 and 41.6% at 5, 10, 30 and 50 min.

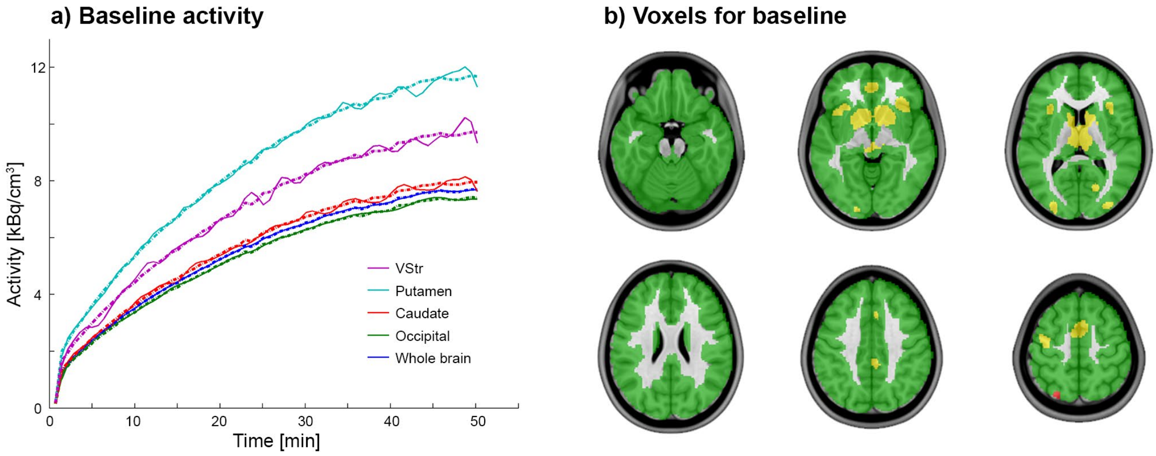

Supplementary figure S2: *Definition and feasibility of the baseline term.* **a)** Baseline time activity curves of a representative subject. Time activity curves were extracted from brain regions of the Harvard-Oxford atlas (solid lines) and fitted with the general linear model (dashed lines). Baseline activity was obtained by subtracting all regressors*β from the raw time activity curve except that of the baseline. Similar to our previous work [7], fitting a single baseline regressor adequately describes the baseline kinetics of various brain regions. **b)** Voxels included (green) and excluded (yellow) to construct the baseline regressor for both women and men (>50% of subjects in each group). A small cluster of 69 voxels was only included for men resulting from different fMRI activation (red), whereas there were no voxels solely included for women. This resulted in an high overlap of voxels used for women and men with Dice = 0.9998. Brain slices are shown at z = -20, -5, 10, 25, 40 and 55 mm MNI space.

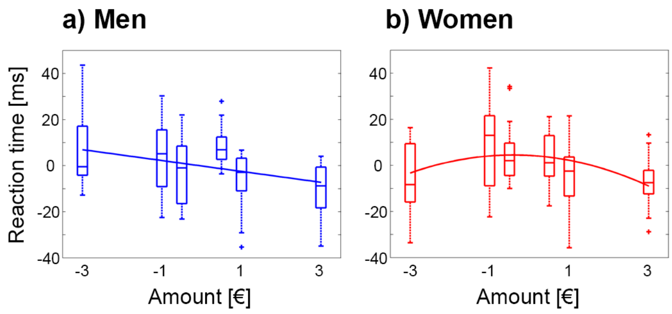

Supplementary figure S3: *Behavioral data separately for men and women (n=18 each)*. Boxplots indicate median reaction times (center line), upper and lower quartiles (box limits) and 1.5*interquartile range (whiskers). The model fits (solid lines) are identical to those shown in figure 2b, but the scaling on the y-axis is different.

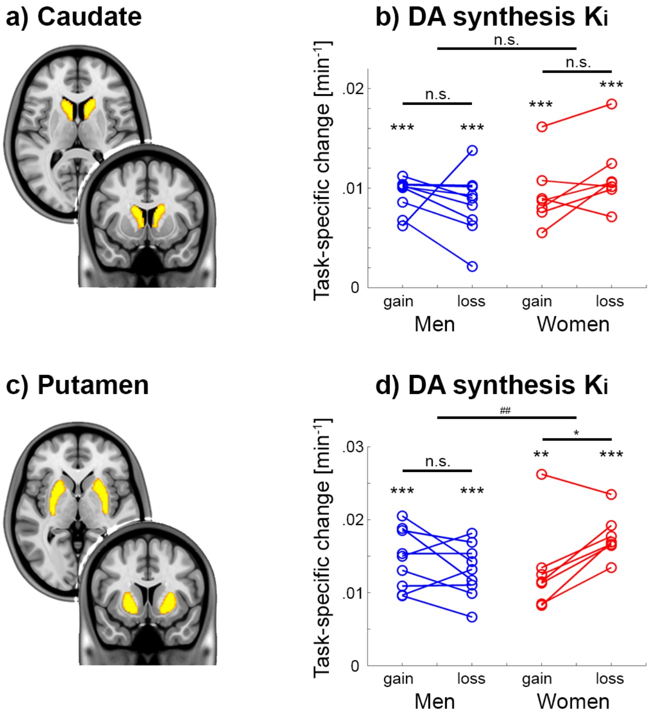

Supplementary figure S4: *Functional PET imaging of task-specific dopamine synthesis*. **a and c)** Regions of interest of the caudate (a) and the putamen (c) from the Harvard-Oxford atlas. **b and d)** Processing of monetary gain and loss resulted in significant increases in dopamine synthesis K_i when compared to baseline (all **$p<0.01$ or ***$p<0.001$). Differences between gain and loss were only significant for the putamen in women (*$p<0.05$), which was driving the sex difference in this region ($^{\#\#}p<0.01$).

SUPPLEMENTARY REFERENCES

1. Pessiglione M, Seymour B, Flandin G, Dolan RJ and Frith CD. Dopamine-dependent prediction errors underpin reward-seeking behaviour in humans. *Nature*. 2006; 442: 1042-5.

2. Rischka L, Gryglewski G, Pfaff S, et al. Reduced task durations in functional PET imaging with [(18)F]FDG approaching that of functional MRI. *Neuroimage*. 2018; 181: 323-30.

3. Huang SC, Yu DC, Barrio JR, et al. Kinetics and modeling of L-6-[18F]fluoro-dopa in human positron emission tomographic studies. *J Cereb Blood Flow Metab*. 1991; 11: 898-913.

4. Doudet DJ, Chan GL, Jivan S, et al. Evaluation of dopaminergic presynaptic integrity: 6-[18F]fluoro-L-dopa versus 6-[18F]fluoro-L-m-tyrosine. *J Cereb Blood Flow Metab*. 1999; 19: 278-87.

5. Kumakura Y, Vernaleken I, Grunder G, Bartenstein P, Gjedde A and Cumming P. PET studies of net blood-brain clearance of FDOPA to human brain: age-dependent decline of [18F]fluorodopamine storage capacity. *J Cereb Blood Flow Metab*. 2005; 25: 807-19.

6. Matsubara K, Watabe H, Kumakura Y, et al. Sensitivity of kinetic macro parameters to changes in dopamine synthesis, storage, and metabolism: a simulation study for [(1)(8)F]FDOPA PET by a model with detailed dopamine pathway. *Synapse*. 2011; 65: 751-62.

7. Hahn A, Gryglewski G, Nics L, et al. Quantification of Task-Specific Glucose Metabolism with Constant Infusion of 18F-FDG. *J Nucl Med*. 2016; 57: 1933-40.
